# A Comparative Accuracy Study of Nasal Soft Tissue Measurements Using a 3D Facial Scanner and Conventional Methods

**DOI:** 10.1155/ijod/6634619

**Published:** 2026-02-16

**Authors:** Negar Ebrahimi, Somayeh Niakan

**Affiliations:** ^1^ Dental Implant Research Center, Dentistry Research Institute, Tehran University of Medical Sciences, Tehran, Iran, tums.ac.ir; ^2^ Department of Prosthodontics, School of Dentistry, Tehran University of Medical Sciences, Tehran, Iran, tums.ac.ir

**Keywords:** dimensional measurement accuracy, face, prosthodontics, software, three-dimensional imaging

## Abstract

**Purpose:**

Analysis of facial morphology is a critical component in craniomaxillofacial prosthetics and surgery, serving purposes such as preoperative diagnosis, postoperative evaluation, and symmetry analysis. This study evaluated the accuracy of a structured‐light 3D facial scanner for measuring facial soft tissues.

**Methods:**

Twenty‐one adult participants were included in this study. A conventional alginate facial impression was obtained from each participant. 3D facial images were also captured using a digital face scanner. Six facial landmarks were identified and recorded on each 3D facial image. The 3D facial scanner and linear caliper measurements on facial casts were employed to measure the distances between landmarks. The accuracy of the 3D facial scanner was evaluated through reliability analysis and paired *t*‐tests.

**Results:**

The mean absolute error of the scanner ranged from 0.18 to 0.44 mm, and the mean relative error ranged from 0.00 to 0.02. Of all the measurements, 69.3% were reproduced within 0.3 mm, and 86.1% within 0.5 mm. The intraclass correlation coefficient (ICC) for all distances was greater than 0.9, demonstrating high consistency. Paired *t*‐test analysis indicated no systematic differences in mean measurements between the scanned images and the facial casts (*p*  > 0.05), except for one distance.

**Conclusion:**

The findings suggest that the face scanner is a precise and reliable alternative for performing maxillofacial measurements.

## 1. Introduction

Soft tissues define the visible shape and movement of the face during interpersonal interactions. Facial morphology analysis plays a vital role in craniofacial and maxillofacial prosthetics and surgery, supporting preoperative diagnosis, postoperative evaluation, and symmetry analysis. Traditionally, facial morphology has been analyzed using two‐dimensional (2D) methods [1, 2]. Recently, advancements in optical scanning technology have elevated facial morphology research from 2D methods to 3D analysis [1, 3].

Until recently, conventional impression materials were used to fabricate maxillofacial prostheses, but these methods posed several challenges requiring significant skill and experience. These challenges include the risk of aspiration during impression taking, distortion of facial soft tissues due to undercuts, patient discomfort, and the technical sensitivity of the materials. The process also involves long working and setting times (5–15 min) and requires skilled technicians to pour the gypsum without voids and to carve and fit the prosthesis to the defect site. When using the traditional approach, the eyes must typically be closed, which makes creating an orbital prosthesis very challenging. In contrast, digitized imaging now allows for quick noncontact 3D facial measurements, producing a functional model only in seconds [4].

Owing to the significant role of accurate facial soft tissue assessment in the fabrication of extraoral prosthesis, with time, many techniques have been developed for the collection of 3D anatomic data, such as computed tomography (CT), cone beam computerized tomography (CBCT), magnetic resonance imaging (MRI), and ultrasound imaging. CT scans involve radiation and high costs [5, 6]. MRI scans are time‐consuming and costly [5]. To address these issues, a few modern 3D imaging techniques have been developed, such as structured‐light scanning and stereophotogrammetry [7]. The primary advantage of 3D facial scans is their capacity to record the patient’s whole face in three dimensions without exposing them to radiation or distortions that might arise with 2D techniques [8]. Structured light acquires the surface of the face via constant light emission, which is subject to deformities because of the unevenness of the scanned surface. The basic principle of 3D scanning with structured light is triangulation. The light is projected in a pattern (usually a series of parallel lines) distorted on the object’s surface. Cameras capture this distortion from multiple angles, and the triangulation calculates the distance to specific points on the object. The three‐dimensional coordinates are then used to reconstruct the object digitally and in great detail [9]. Contrarily, stereophotogrammetry uses a multicamera system to concurrently take two or more photos of the same object from various angles. This method has benefits such as avoiding uncontrollable face movements and facial expressions that could compromise the scan’s accuracy [10, 11].

Prostheses can be digitally designed by mirroring the healthy side of the face. Furthermore, coordinating the prosthesis color with the patient’s skin tone becomes more efficient with digital tools, simplifying the process. A digital workflow facilitates the creation of symmetrical designs and ensures proper shaping of the prosthesis to harmonize with the patient’s facial features [4]. Consequently, prostheses can be fabricated more efficiently, with reduced time and minimal effort. However, the higher cost of the required software and equipment remains a significant consideration. A low‐cost and accessible approach to facial scanning involves using smartphone‐based scanning technologies. Advances in smartphone camera quality have made them suitable for clinical facial scans [10, 12].

The accuracy of a facial scanner means the closeness of the data measured by the scanner and the true value [13]. All scanners have a nominal accuracy (NA), determined by measuring standard geometries in the factory. However, several recent studies have reported that there are indeed differences between the NA and the practical accuracy (PA) of face scanners, as the scanned object (the face of a real person) has a more complex shape and texture than a standard model [14, 15]. Freedom F Face Scanner (DOF Inc., Seoul, South Korea) is a 3D scanner with a structured light mechanism. This scanner operates at a rated voltage of DC 5 V/0.6 A and requires 3 W of power. Its depth resolution is 640 × 480 (VGA), and it captures depth at distances between 0.2 m and 1.5 m. The device outputs data in OBJ format and uses infrared (IR) light triangulation as its measurement method [16]. This study aims to assess the accuracy and reliability of the Freedom F Face Scanner (DOF Inc., Seoul, South Korea) in measuring facial soft tissue distances. Although several previous studies have evaluated the accuracy of 3D facial scanners [4], few have focused on high‐resolution extraoral scanners like the Freedom F in assessing facial soft tissue landmarks using direct comparison with conventional plaster casts. Our study is among the first to quantify measurement error across multiple facial regions using a standardized landmark transfer method and to classify errors based on clinical relevance.

## 2. Materials and Methods

### 2.1. Sample

Sample size estimation was performed using PASS 2025 (Power Analysis and Sample Size Software, NCSS, LLC, Kaysville, Utah, USA) to determine the number of participants required to detect a clinically meaningful difference of 0.3 mm between paired measurements from 3D facial scans and plaster casts. The 0.3 mm threshold was selected based on evidence from a recent systematic review [4], which identified 0.3 mm as a clinically meaningful accuracy limit for facial measurements; this is further detailed in the Discussion section.

Based on a standard deviation of paired differences of 0.3 mm, a two‐sided significance level (α) of 0.05, and a desired power of 90%, the analysis indicated that a minimum of 13 participants would be sufficient. To ensure robust statistical reliability, this study included 21 adult participants (9 males and 12 females).

### 2.2. Ethics

All participants provided informed consent before participation. Ethical approval was obtained from the local ethics committee and institutional review board (IR.TUMS.DENTISTRY.REC.1399.081).

### 2.3. Facial Impression Procedure and Plaster Cast Fabrication

Conventional alginate impressions were taken of each participant’s nasal region and upper lip, extending to the commissures. Participants were positioned flat on a dental chair during the procedure. To prevent adhesion, hair was secured with a hairband, and eyebrows were greased with Vaseline (Saj, Karaj, Iran). Small plastic tubes were inserted into the nostrils to maintain breathing. Fast‐setting alginate (Chromogel alginate, Marlik, Tehran, Iran) was applied to the nasal and upper lip regions. Curved paper clips were embedded in the alginate as retention aids for the reinforcement layer. A layer of fast‐setting plaster (Adentatec Superhard Plaster, Wörth am Rhein, Germany) was then applied to enhance stability. Once set, the plaster‐reinforced impression was carefully removed. The impressions were then filled with hard plaster (Moldano, Atlantic Plaster, Tehran, Iran) to create detailed facial casts.

### 2.4. 3D Facial Scanning

Participants’ facial surfaces were scanned using the Freedom F face scanner (DOF Inc., Seoul, South Korea). Participants were asked to maintain a natural head position by looking straight ahead at eye level, with the Frankfort horizontal plane parallel to the floor, and their lips relaxed and naturally closed. This positioning was consistent across all participants and was monitored by the operator to ensure reproducibility.

Measurements on the 3D scans were performed using Exocad’s Dental CAD software (Exocad GmbH, Align Technology, Inc). The handheld scanner was connected to a Surface laptop (Microsoft Corporation, Redmond, Washington). The operator completed each scan from left to right within 10 s.

### 2.5. Landmark Identification and Measurement

**Figure 1:**
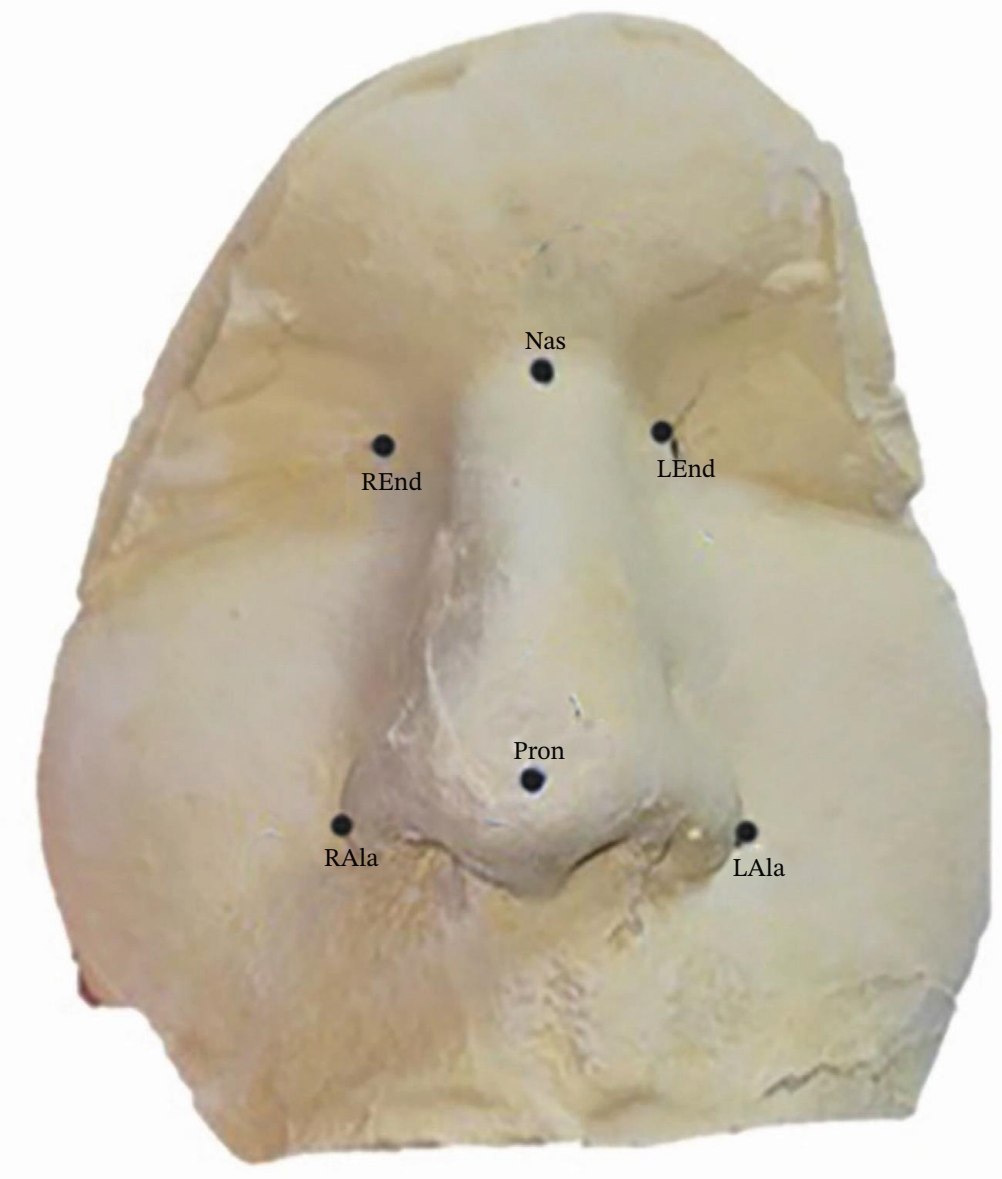
Landmarks evaluated: Pron (pronasale: most protruded point of the apex nasi), LAla and RAla (left alar and right alar: most lateral point of the alar contour), LEnd and REnd (endocanthion: inner commissure of eye fissure), and Nas (nasion: deepest point of nasal bridge).

Six anatomical landmarks were identified on both the facial scans and plaster casts, covering the nasal area and upper lip up to the commissures. Figure 1 illustrates a molded cast with these landmarks:

• Pron (pronasale): most protruded point of the nasal apex
• LAla and RAla (left and right alar): most lateral points of the alar contour
• LEnd and REnd (left and right endocanthion): inner commissures of the eye fissures
• Nas (nasion): deepest point of the nasal bridge

To align the cast landmarks with those on the scanned images, researchers used a copying pencil to mark them on participants’ faces before taking impressions. These markings transferred first to the alginate impression and then to the plaster cast. In the 3D scan files, landmarks were identified based on these transferred markings.

Eleven interlandmark distances were measured: Pron‐RAla, Pron‐LAla, Pron‐REnd, Pron‐LEnd, RAla‐LAla, RAla‐REnd, LAla‐LEnd, REnd‐LEnd, Pron‐Nas, Nas‐REnd, and Nas‐LEnd. The shortest distance between two reference points was recorded for both methods. To minimize variability in landmark identification, all measurements were performed by a single calibrated examiner who was trained and experienced in both anatomical landmark localization and the use of the measurement software. This approach was adopted to ensure methodological consistency and eliminate interobserver variability. Measurement errors were calculated as the difference between the two methods and categorized as follows: <0.3 mm, <0.5 mm, and >0.5 mm.

### 2.6. Statistical Analysis

Data were analyzed using SPSS (IBM SPSS Statistics for Windows, Version 27.0). The Shapiro‐Wilk test assessed data normality. Absolute errors were calculated by subtracting scanner‐derived measurements from plaster cast measurements. Relative accuracy errors were determined by dividing the absolute error by the plaster cast measurement.

Agreement between methods was evaluated using the intraclass correlation coefficient (ICC) and visualized with Bland–Altman plots. A paired *t*‐test was conducted to assess significant differences between the two measurement methods. The significance level (type I error) was set at 0.05.

## 3. Results

**Figure 2:**
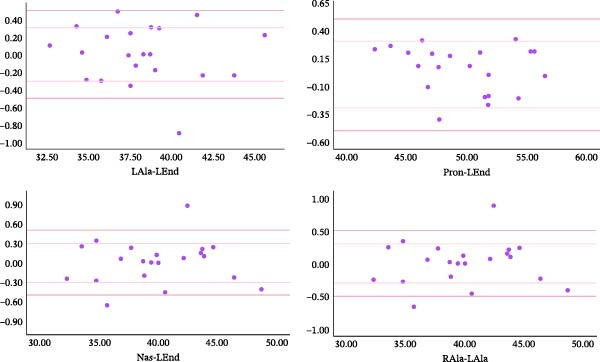
Bland‐Altman plots showing the reproducibility of LAla‐LEnd, Pron‐LEnd, Nas‐LEnd, and RAla‐LAla distances (Vertical axes show the difference between the cast and scanner measurements. Horizontal axes show the average measurements).

**Figure 3:**
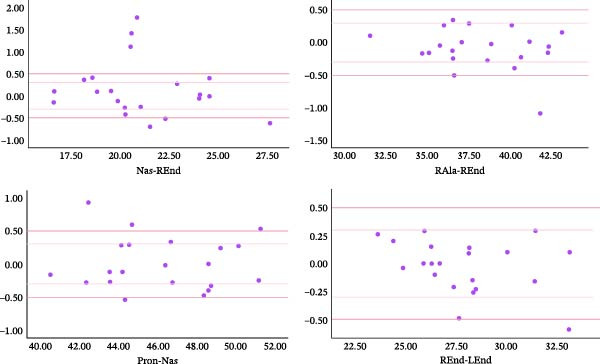
Bland‐Altman plots showing the reproducibility of Nas‐REnd, RAla‐REnd, Pron‐Nas, and REnd‐LEnd distances (Vertical axes show the difference between the cast and scanner measurements. Horizontal axes show the average measurements).

**Figure 4:**
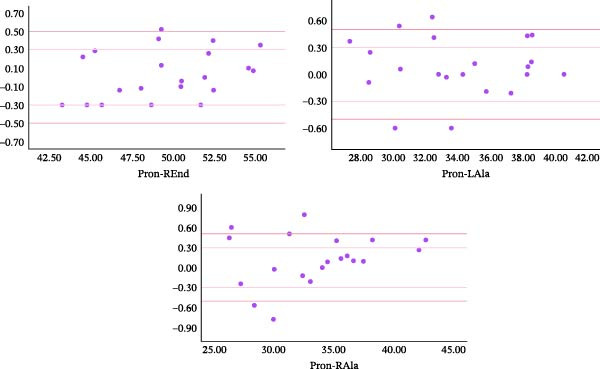
Bland‐Altman plots showing the reproducibility of Pron‐REnd, Pron‐LAla, and Pron‐RAla distances (Vertical axes show the difference between the cast and scanner measurements. Horizontal axes show the average measurements).

According to the Shapiro‐Wilk analysis, all the measured values by the scanner and on the cast were normal variables. The mean absolute error of the scanner ranged from 0.18 to 0.44. The mean relative error of the scanner ranged from 0.00 to 0.02 (Table 1). Absolute errors of scanner measurement were categorized into three ranges: less than 0.3 mm, between 0.3 mm and 0.5 mm, and more than 0.5 mm. Of all the measurements, 69.26%, 16.88%, and 13.85% showed errors less than 0.3 mm, between 0.3 mm and 0.5 mm, and more than 0.5 mm, respectively. The Bland–Altman plots of the distances are shown in Figures 2–4. These figures show the frequency distribution of measurements relative to the two limits 0.3 and 0.5.

**Table 1:** Mean measurements of lengths, absolute errors, and relative errors.

Inter‐landmark distances	Mean (±SD) of scanner measurements	Mean (±SD) of reference (cast) measurements	Mean absolute error (mm) (±SD)	Mean relative error (%) (±SD)
Pron‐RAla	33.56 (±4.66)	33.44 (±4.57)	0.30 (±0.24)	0.01 (±0.01)
Pron‐LAla	34.1 (±3.9)	34.00 (±3.95)	0.25 (±0.22)	0.01 (±0.01)
Pron‐REnd	49.6 (±3.6)	49.53 (±3.52)	0.23 (±0.14)	0.00 (±0.00)
Pron‐LEnd	49.79 (±4.07)	49.73 (±4.11)	0.19 (±0.10)	0.00 (±0.00)
RAla‐LAla	39.92 (±4.40)	39.91 (±4.37)	0.25 (±0.22)	0.01 (±0.01)
RAla‐REnd	38.22 (±3.01)	38.32 (±3.08)	0.24 (±0.24)	0.01 (±0.01)
LAla‐LEnd	38.21 (±3.17)	38.21 (±3.20)	0.25 (±0.20)	0.01 (±0.01)
REnd‐LEnd	27.90 (±2.62)	27.93 (±2.70)	0.18 (±0.15)	0.01 (±0.01)
Pron‐Nas	46.21 (±3.08)	46.20 (±3.09)	0.32 (±0.21)	0.01 (±0.00)
Nas‐REnd	21.21 (±2.75)	21.07 (±2.87)	0.44 (±0.47)	0.02 (±0.02)
Nas‐LEnd	20.30 (±1.79)	20.05 (±1.97)	0.42 (±0.38)	0.02 (±0.02)

The ICC between the scanner and cast measurements is shown in Table 2. All distances demonstrated high consistency (ICC more than 0.9). The paired *t*‐test revealed no statistically significant difference between the scanner and cast measurements for all the distances (*p* > 0.05), except Nas‐LEnd (*p* = 0.03) (Table 3). In other words, the face scanner did not systematically differ from the cast in terms of the overall mean measurements.

**Table 2:** Intraclass correlation coefficient showing the agreement between the measurements of the two groups.

Inter‐landmark distances	Intraclass correlation coefficient (95% confidence interval)	*p*‐value
Pron‐RAla	0.998 (0.996, 0.999)	<0.0001
Pron‐LAla	0.998 (0.996, 0.999)	<0.0001
Pron‐REnd	0.998 (0.996, 0.999)	<0.0001
Pron‐LEnd	0.999 (0.998, 1.000)	<0.0001
RAla‐LAla	0.999 (0.996, 0.999)	<0.0001
RAla‐REnd	0.997 (0.993l, 0.999)	<0.0001
LAla‐LEnd	0.997 (0.993, 0.999)	<0.0001
REnd‐LEnd	0.998 (0.995, 0.999)	<0.0001
Pron‐Nas	0.996 (0.990, 0.998)	<0.0001
Nas‐REnd	0.987 (0.968, 0.995)	<0.0001
Nas‐LEnd	0.982 (0.955, 0.993)	<0.0001

**Table 3:** Results of *t*‐test showing significant differences between two groups.

Statistical values	Pron‐RAla	Pron‐LAla	Pron‐REnd	Pron‐LEnd	RAla‐LAla	RAla‐REnd	LAla‐LEnd	REnd‐LEnd	Pron‐Nas	Nas‐REnd	Nas‐LEnd
Mean difference (±SD)	0.11 (±0.38)	0.08 (±0.33)	0.03 (±0.27)	0.05 (±0.21)	0.01 (±0.33)	−0.10 (±0.32)	0.00 (±0.33)	−0.03 (±0.24)	0.01 (±0.39)	0.14 (±0.63)	0.26 (±0.50)
*t*‐statistic	1.39	1.18	0.58	1.19	0.12	−1.44	0.00	−0.56	0.12	1.01	2.38
*p*‐value	0.18	0.25	0.56	0.25	0.91	0.16	1.00	0.58	0.91	0.32	0.03

## 4. Discussion

Accurate measurement of facial dimensions is critical for the fabrication of maxillofacial prostheses [4, 17]. Our study evaluated the accuracy of the Freedom F Face Scanner (DOF Inc., Seoul, South Korea) compared to direct measurements on facial casts with a digital vernier caliper, focusing on the landmarks relevant to nasal prostheses. We measured 11 distances on the nasal region.

Except for the Nas‐LEnd distance, mean measurements did not differ systematically from cast measurements. The ICC confirmed high reliability across all landmarks. These results support the practical utility of the structured‐light scanner (Freedom F Face Scanner, DOF Inc., Seoul, South Korea). Recent literature further supports and contextualizes our findings. Comparative studies of stereophotogrammetry and structured‐light scanners have shown that while stereophotogrammetry systems generally provide superior accuracy, structured‐light scanners offer efficient acquisition times and favorable user‐friendliness, making them clinically practical for routine use [18]. Emerging smartphone‐based structured‐light technologies also demonstrate promising agreement with stereophotogrammetry, though they require longer acquisition and processing times and greater operator attention. Because the smartphone applications are more affordable and portable, any practitioner can use them [19].

The findings of the current study are in line with previous studies that assessed the accuracy of 3D scanning technologies; Toma et al. [3] evaluated a laser‐scan 3D imaging system (Konica/Minolta). Similar to the current investigation, most of the facial landmarks were reproducible to less than 1 mm, which was considered clinically acceptable. In Toma’s study, most poorly reproducible coordinates were around the eyes due to their complex geometry. Landmarks on well‐defined borders were more reproducible than those on curved surfaces [3]. In the current study, Nas‐LEnd distance, which is related to the left eye, showed a significant difference between manual and scanner measurements, which may be due to poorly defined borders, as Toma et al. [3] stated. This distance demonstrated a mean absolute error of 0.42 mm (±0.38 mm), which falls within our predefined acceptable error threshold of 0.5 mm. Although this value exceeded the ideal error margin of 0.3 mm, it remains clinically acceptable. The slightly higher error likely reflects the complexity of identifying nasion and endocanthion, located in areas with subtle soft tissue contours. Likewise, Germec‐Cakan et al. noted that laser scanners struggle with deep or curved anatomical regions, supporting the view that subtle contours and recessed locations affect scanner accuracy [20].

A recent systematic review compared interlandmark distances measured virtually and directly on the face. It found no significant differences between caliper and scanning methods. This review asserts that no ideal equipment or software is currently available to measure facial distances and argues that the accuracy threshold is 0.3 mm. A layperson could notice a difference between a silicone prosthesis and skin if it is greater than 0.3 mm. Several scanners have a NA of 0.2–0.3 mm. However, even 0.3 mm is regarded as a significant difference in facial prosthetics. Nevertheless, it is advised to use scanning systems since the drawbacks of the conventional impression‐taking technique unquestionably exceed the disadvantages of facial scanners [4]. We considered an absolute error of 0.5 mm or less as acceptable and an error of 0.3 mm or less as ideal. More than two‐thirds of the measurements were reproduced to less than 0.3 mm. The threshold of 0.5 mm was selected based on values reported in previous studies [3].

We used cast‐based measurements as reference—other studies used CBCT, direct facial caliper, or industrial scanners [14, 20, 21]. One of the positive points of the current study was the implementation of the study in a real clinical setting. Some similar studies used models instead of human faces or mouths [21, 22]. An in vitro study by Amornvit and Sanohkan [22] assessed four different face‐scanning devices and compared their accuracy with direct measurements taken using a vernier caliper. Master face models were printed and scanned with four scanners, five times. Scanning length, pattern, and technology significantly affected the accuracy. These scanners were not suggested for assessing a depth of more than 2 mm [22].

Clinical use of 3D scanners depends on affordability, patient satisfaction, time efficiency, and ease of use. Sa’ Gomes et al. [23] evaluated the Artec Eva scanner for both accuracy and time efficiency. Measurements with the scanner were compared to direct facial measurements with a caliper—both with and without premarked facial landmarks. Similar to our study, one trained operator performed all the measurements. Their study confirmed that marking facial landmarks before scanning reduces the time needed to complete the measurements. The scanner required more time than the direct measurement method. This highlights the need for further advancements in scanner technology to enable faster image capture and processing [23]. The results of Sa’ Gomes’ study [23] support previous research, showing strong replicability both within and between methods for unmarked and marked landmarks in most of the 11 measurements analyzed, suggesting that facial scanning is a valid assessment method. Overall, accuracy approximately doubled when the landmarks were premarked [23]. This was the reason we marked all the landmarks on the face in our study.

In many areas of dentistry, 3D facial scanning is now routinely used for diagnosis, treatment planning, and postoperative evaluation across specialties such as maxillofacial surgery, prosthodontics, and orthodontics [24]. Several factors have contributed to the growing use of facial scanning in clinical dentistry. Most notably, the technology enables objective, quantitative evaluation of facial morphology [25–28]. In addition, recent advancements in portable devices and software have made facial scanning more widely available than ever [29, 30]. The capability to generate detailed 3D models supports more precise clinical decision‐making and improves patient engagement [31, 32]. Despite its growing clinical potential, several barriers still hinder the widespread adoption of 3D facial scanning in dentistry. Elevated expenses regarding equipment, software, and maintenance, accessibility, and the absence of standardized scanning protocols lead to variability across research and clinical practice [29, 30].

Landmark reproducibility depends on the measurement technique, examiner skill, software familiarity, anatomical surface shape, image quality, and clear definitions of landmarks [20]. A potential limitation of this study is the absence of intra‐ and interobserver reliability assessment. However, to reduce variability, all measurements were conducted by a single calibrated examiner under standardized conditions. While this approach ensured internal consistency, it may limit the generalizability of reproducibility across clinical settings, as different operators may identify landmarks slightly differently. Establishing standardized landmark identification protocols and validating reproducibility across multiple operators would therefore be an important next step. Future studies involving multiple examiners or repeated measurements over time are recommended to validate the reproducibility of landmark identification. Another limitation of this study is that measurements were confined to the nasal region. Conventional impression materials are technically challenging to use over large facial areas. Therefore, we focused on a smaller region to improve precision since the primary goal was to evaluate the scanner’s accuracy in clinically relevant nasal areas. Nonetheless, this restriction limits the applicability of our results to comprehensive craniofacial analyses, where variability may be greater across broader regions. Future investigations should therefore assess the accuracy of facial scanners in both nasal and full‐facial contexts.

In this study, reference lengths were measured directly on casts, which may have included some errors, though within an acceptable range. The manual method of taking facial impressions is simple and cost‐effective, but it has some limitations. Soft tissue distortion during alginate application, along with material shrinkage or expansion during setting and casting, can lead to minor inaccuracies that may affect measurement results [20]. However, this issue is controversial. For instance, one study showed that there is no significant difference between cast measurements and CBCT, and both methods are accurate for facial 3D analysis [33]. Anthropometrical interlandmark measurement might have errors. For instance, the soft tissues of the face might not be in a resting position during measurement. In addition, the sample was selected from a healthy adult population, which does not accurately represent individuals who typically receive facial prosthetics. Therefore, future studies should be conducted on patients requiring maxillofacial prostheses and should consider using higher‐precision reference standards, such as high‐resolution CT or CBCT imaging, to further validate the accuracy of 3D facial scanners in clinically relevant settings.

## 5. Conclusion

This study provides one of the first accuracy assessments of the Freedom F structured‐light 3D facial scanner for nasal soft tissue measurements using direct plaster cast measurements as the reference standard. The scanner reproduced more than two‐thirds of all interlandmark distances within the ideal clinical threshold of 0.3 mm and more than 80% within 0.5 mm, demonstrating its suitability for dimensions relevant to nasal and perioral prostheses. The error‐range classification used in this study offers a clinically interpretable framework for evaluating scanner performance in prosthetic design. While facial scanners are known to be generally reliable, our results provide new, scanner‐specific evidence for accuracy in the nasal region, particularly when landmarks are premarked and located in areas with complex soft tissue contours. Further studies, including broader anatomical regions, larger sample sizes, prosthesis fabrication outcomes, and multiobserver reproducibility, are recommended to confirm these findings and support clinical translation.

## Funding

This research received no specific grant from any funding agency in the public, commercial, or not‐for‐profit sectors.

## Conflicts of Interest

The authors declare no conflicts of interest.

## Data Availability

The data that support the findings of this study are available from the corresponding author upon reasonable request.

## References

[1] Berssenbrügge P. , Berlin N. F. , and Kebeck G. , et al.2D and 3D Analysis Methods of Facial Asymmetry in Comparison, Journal of Cranio-Maxillofacial Surgery. (2014) 42, no. 6, e327–e334, 10.1016/j.jcms.2014.01.028, 2-s2.0-84905678288. 24507934

[2] Kim J.-Y. , Jung H.-D. , Jung Y.-S. , Hwang C.-J. , and Park H.-S. , A Simple Classification of Facial Asymmetry by TML System, Journal of Cranio-Maxillofacial Surgery. (2014) 42, no. 4, 313–320, 10.1016/j.jcms.2013.05.019, 2-s2.0-84899941466. 23810748

[3] Toma A. M. , Zhurov A. , Playle R. , Ong E. , and Richmond S. , Reproducibility of Facial Soft Tissue Landmarks on 3D Laser-Scanned Facial Images, Orthodontics & Craniofacial Research. (2009) 12, no. 1, 33–42, 10.1111/j.1601-6343.2008.01435.x, 2-s2.0-64749091184. 19154273

[4] König J. , Czumbel L. M. , and Szabó B. , et al.Current Status of Optical Scanning in Facial Prosthetics: A Systematic Review and Meta-Analysis, Journal of Prosthodontic Research. (2024) 68, no. 1, 1–11, 10.2186/jpr.JPR_D_22_00221. 37286516

[5] Goyal M. K. , Goyal S. , and Dhanasekar B. , Modern Trends in Modeling of Extra-Oral Defects, Indian Journal of Dental Research. (2014) 25, no. 1, 128–132, 10.4103/0970-9290.131170, 2-s2.0-84899765486. 24748317

[6] Tasopoulos T. , Kouveliotis G. , Polyzois G. , and Karathanasi V. , Fabrication of a 3D Printing Definitive Obturator Prosthesis: a Clinical Report, Acta Stomatologica Croatica. (2017) 51, no. 1, 53–59, 10.15644/asc51/1/7, 2-s2.0-85016181149. 28740271 PMC5506255

[7] Nayar S. and Mahadevan R. , A Paradigm Shift in the Concept for Making Dental Impressions, Journal of Pharmacy and Bioallied Sciences. (2015) 7, no. Suppl 1, S213–S215, 10.4103/0975-7406.155910, 2-s2.0-84928940037. 26015714 PMC4439674

[8] Pellitteri F. , Scisciola F. , Cremonini F. , Baciliero M. , and Lombardo L. , Accuracy of 3D Facial Scans: A Comparison of Three Different Scanning System in an In Vivo Study, Progress in Orthodontics. (2023) 24, no. 1, 10.1186/s40510-023-00496-x, 44. 38143253 PMC10749289

[9] Al-Anezi T. , Khambay B. , Peng M. J. , O’Leary E. , Ju X. , and Ayoub A. , A New Method for Automatic Tracking of Facial Landmarks in 3D Motion Captured Images (4D), International Journal of Oral and Maxillofacial Surgery. (2013) 42, no. 1, 9–18, 10.1016/j.ijom.2012.10.035, 2-s2.0-84872006767. 23218511

[10] Akan B. , Akan E. , Şahan A. O. , and Kalak M. , Evaluation of 3D Face-Scan Images Obtained by Stereophotogrammetry and Smartphone Camera, International Orthodontics. (2021) 19, no. 4, 669–678, 10.1016/j.ortho.2021.08.007. 34544662

[11] Gibelli D. , Pucciarelli V. , and Caplova Z. , et al.Validation of a Low-Cost Laser Scanner Device for the Assessment of Three-Dimensional Facial Anatomy in Living Subjects, Journal of Cranio-Maxillofacial Surgery. (2018) 46, no. 9, 1493–1499, 10.1016/j.jcms.2018.06.009, 2-s2.0-85049306612. 30196857

[12] Kim A. , Gu D. , and Chandiramani R. , et al.Accuracy and Reliability of Digital Craniofacial Measurements Using a Small-Format, Handheld 3D Camera, Orthodontics & Craniofacial Research. (2018) 21, no. 3, 132–139, 10.1111/ocr.12228, 2-s2.0-85050119566. 29863289

[13] Petrides G. , Clark J. A. R. , Low H. , Lovell N. , and Eviston T. J. , Three-Dimensional Scanners for Soft-Tissue Facial Assessment in Clinical Practice, Journal of Plastic, Reconstructive & Aesthetic Surgery. (2021) 74, no. 3, 605–614, 10.1016/j.bjps.2020.08.050. 33082078

[14] Zhao Y.-J. , Xiong Y.-X. , Wang Y. , and Bencharit S. , Three-Dimensional Accuracy of Facial Scan for Facial Deformities in Clinics: A New Evaluation Method for Facial Scanner Accuracy, PLOS ONE. (2017) 12, no. 1, 10.1371/journal.pone.0169402, 2-s2.0-85008660975. PMC5215889 28056044

[15] Zhao Y. , Xiong Y. , Yang H. , and Wang Y. , Evaluation of Measurement Accuracy of Three Facial Scanners Based on Different Scanning Principles, Journal of Peking University. Health Sciences. (2014) 46, no. 1, 76–80. 24535353

[16] D. O. F. Freedom F. Dental Face Scanner , 2025, (Accessed 7/27/2025.) https://minosec.com/index.php?route=product/product&product_id=374.

[17] Abduo J. and Elseyoufi M. , Accuracy of Intraoral Scanners: A Systematic Review of Influencing Factors, The European Journal of Prosthodontics and Restorative Dentistry. (2018) 26, no. 3, 101–121, 10.1922/EJPRD_01752Abduo21, 2-s2.0-85055614104. 29989757

[18] Jindanil T. , Ponbuddhichai R. , and Massant C. , et al.Three-Dimensional Facial Imaging: A Comparative Assessment of the Clinical Applicability of State-of-the-Art Technologies for Three-Dimensional Facial Imaging, International Journal of Dentistry. (2025) 2025, no. 1, 10.1155/ijod/8822293, 8822293. 40401237 PMC12094855

[19] D‘Ettorre G. , Farronato M. , Candida E. , Quinzi V. , and Grippaudo C. , A Comparison Between Stereophotogrammetry and Smartphone Structured Light Technology for Three-Dimensional Face Scanning, The Angle Orthodontist. (2022) 92, no. 3, 358–363, 10.2319/040921-290.1. 35015071 PMC9020391

[20] Germec-Cakan D. , Canter H. I. , Nur B. , and Arun T. , Comparison of Facial Soft Tissue Measurements on Three-Dimensional Images and Models Obtained With Different Methods, Journal of Craniofacial Surgery. (2010) 21, no. 5, 1393–1399, 10.1097/SCS.0b013e3181ec6976, 2-s2.0-77957341194. 20856027

[21] Mangano F. G. , Hauschild U. , Veronesi G. , Imburgia M. , Mangano C. , and Admakin O. , Trueness and Precision of 5 Intraoral Scanners in the Impressions of Single and Multiple Implants: A Comparative In Vitro Study, BMC Oral Health. (2019) 19, no. 1, 101. 31170969 10.1186/s12903-019-0792-7 PMC6555024

[22] Amornvit P. and Sanohkan S. , The Accuracy of Digital Face Scans Obtained From 3D Scanners: An In Vitro Study, International Journal of Environmental Research & Public Health. (2019) 16, no. 24, 10.3390/ijerph16245061, 5061. 31842255 PMC6950499

[23] de Sá Gomes C. F. , Libdy M. R. , and Normando D. , Scan Time, Reliability and Accuracy of Craniofacial Measurements Using a 3D Light Scanner, Journal of Oral Biology and Craniofacial Research. (2019) 9, no. 4, 331–335, 10.1016/j.jobcr.2019.07.001, 2-s2.0-85069679367. 31388482 PMC6669702

[24] Takahiro S. , Yuichi M. , Akina T. , Tsuyoshi T. , and Takeshi M. , Facial Scans in Clinical Dentistry and Related Research: A Scoping Review, Cureus. (2025) 17, no. 4. 10.7759/cureus.81662 PMC12049179 40322431

[25] Carvalho P. E. G. , Ortega A. D. O. , Maeda F. A. , da Silva L. H. , Carvalho V. G. G. , and Torres F. C. , Digital Scanning in Modern Orthodontics, Current Oral Health Reports. (2019) 6, no. 4, 269–276, 10.1007/s40496-019-00235-4.

[26] Matsuo M. , Mine Y. , Kawahara K. , and Murayama T. , Accuracy Evaluation of a Three-Dimensional Model Generated From Patient-Specific Monocular Video Data for Maxillofacial Prosthetic Rehabilitation: A Pilot Study, Journal of Prosthodontics. (2020) 29, no. 8, 712–717, 10.1111/jopr.13219. 32583571

[27] Lee G. H. , Park J. H. , Park J. J. , Lee K. C. , Lee S. M. , and Moon D. , Key Considerations for Efficient 3-Dimensional Data Integration With a Face Scanner in the Digital Era: Product Review of the Hybrid Face Scanner, 2024, Elsevier.

[28] Tabira K. , Kawaguchi R. , and Mine Y. , et al.An In Vitro Study of Digital Impressions and Three-Dimensional Printed Models of Orbital Defects Using Mobile Devices and Monoscopic Photogrammetry, Journal of Oral Science. (2023) 65, no. 2, 127–130, 10.2334/josnusd.22-0461. 36990757

[29] Singh P. , Bornstein M. M. , Hsung R. T..-C. , Ajmera D. H. , Leung Y. Y. , and Gu M. , Frontiers in Three-Dimensional Surface Imaging Systems for 3D Face Acquisition in Craniofacial Research and Practice: An Updated Literature Review, Diagnostics. (2024) 14, no. 4, 10.3390/diagnostics14040423, 423. 38396462 PMC10888365

[30] Jindanil T. , Xu L. , Fontenele R. C. , Perula M. C. D. L. , and Jacobs R. , Smartphone Applications for Facial Scanning: A Technical and Scoping Review, Orthodontics & Craniofacial Research. (2024) 27, no. S2, 65–87, 10.1111/ocr.12821. 38842250 PMC11654360

[31] Lahoud P. , Jacobs R. , Boisse P. , EzEldeen M. , Ducret M. , and Richert R. , Precision Medicine Using Patient-Specific Modelling: State of the Art and Perspectives in Dental Practice, Clinical Oral Investigations. (2022) 26, no. 8, 5117–5128, 10.1007/s00784-022-04572-0. 35687196

[32] Isikay I. , Cekic E. , Baylarov B. , Tunc O. , and Hanalioglu S. , Narrative Review of Patient-Specific 3D Visualization and Reality Technologies in Skull Base Neurosurgery: Enhancements in Surgical Training, Planning, and Navigation, Frontiers in Surgery. (2024) 11, 10.3389/fsurg.2024.1427844, 1427844. 39081485 PMC11287220

[33] Xu Y. , Li J. , Zhao S. , Shi B. , Zheng Q. , and Wang Y. , Accuracy of a Plastic Facial Cast Fabricated With a Custom Tray in Comparison With Cone Beam Computed Tomography, Oral Surgery, Oral Medicine, Oral Pathology and Oral Radiology. (2014) 117, no. 3, e238–e245, 10.1016/j.oooo.2012.04.021, 2-s2.0-84893959468. 22939320

